# A Randomized, Double-Blind, Placebo-Controlled Evaluation of the Safety and Efficacy of Wild Sage Metabolites in Preventing Snoring, Improving Sleep, and Activating Alpha Wave Brain Frequencies in Healthy Adults

**DOI:** 10.7759/cureus.22714

**Published:** 2022-02-28

**Authors:** Yuki Ikeda, Mizuho Nasu

**Affiliations:** 1 Microbiology, Innovation Labo Research Center, Tokyo, JPN; 2 Pharmacology, Innovation Labo Research Center, Tokyo, JPN

**Keywords:** stress, alpha waves, gut microbiota, sleeping, snoring, wild sage metabolites

## Abstract

Upper airway problems and stress can cause sleep disorders. The present study was designed to evaluate the efficacy and safety of wild sage metabolites (WSM) for snoring treatment and alpha wave activation in healthy men and women. A total of 42 subjects compliant with inclusion criteria were randomly assigned to one of the two groups, viz. Group 1-WSM and Group 2-Placebo, using a simple randomization process. Consumption of WSM by healthy men and women resulted in the decrease of Pittsburgh Sleep Quality Index (PSQI) global score by 29%, improved assessment by their sleep mates, and increased alpha brain wave by 55%. In conclusion, medication with WSM resulted in signiﬁcant reduced snoring, stress, and improved sleep quality after 30 days, with a good tolerance among subjects. No side effects or adverse events were reported during the study. Hence, WSM at 450 mg/day could be recommended as an effective agent for snoring treatment, improving the quality of sleep, and stress reduction.

## Introduction

Upper airway problems can cause sleep disorders. The clinical presentation in patients can be observed in many different forms, from simple snoring to obstructive sleep apnea syndrome (OSAS). An airway constriction that occurs because of relaxation of the soft tissue anatomy in the oropharyngeal region increases the speed of airflow during sleeping. Air that quickly passes through narrow passage results in loud sounds due to the vibrating unsupported tissues of the upper airway [1].

Surgeries, such as septorhinoplasty, that improve nasal patency have an important role in the treatment of snoring. However, septorhinoplasty does not provide complete correction of snoring in conditions such as sagging of the soft palate and bulking of the soft palate-uvula tissue and/or the tongue [2]. Therefore, elimination of upper airway problems with correction of current hypotonia in the muscles of the soft palate is required in the treatment of snoring.

There are many factors and mechanisms, both internal and external, involved in the regulation and quality of sleep. Even though the central nervous system plays a main role within the internal ones, it is worth noting that the role of several substances and metabolites such as vitamin D that, beyond its well-known contribution to the bone and mineral metabolism, has been shown to exert regulatory effects in the sleep-wake cycle modulation. There is evidence proving that lower levels in the organism of such metabolites in healthy adults lead to a higher likelihood of sleep disorders [3-4].

Innovation Labo developed a complex of wild sage metabolites (WSM), containing a complex of metabolites from natural fermentation which not only stimulates muscle cell differentiation thus reducing hypotonia in the upper airway muscles, but also increases the activity of the alpha frequency band that represents the relaxing effect on the central nervous system, resulting in decreasing of stress and, therefore, improving the quality of sleep. Hence, supplementation or intake of WSM might improve sleep, according to prior published evidence.

The mechanisms through which WSM is related to sleep disorders are not yet well-defined, however, it is believed that some specific receptors are relevant in such a correlation since they are extensively found in the brain, particularly in essential structures for sleep regulation such as the hypothalamus or the prefrontal cortex. Alternatively, WSM includes determinant molecules known to play a role as immunomodulators, and therefore, their deficiency causes the release of inflammatory substances, some of which regulate sleep (prostaglandin D2 and various cytokines) [5].

Apart from modulating sleep regulation, sleep quality is also influenced by the metabolites of wild sage through their involvement in regards to the upper airway muscle tone. It has been demonstrated that cellular proliferation and differentiation can be altered using several mechanisms, most of which imply the enhancement or suppression of transcription of cell-growth-regulating genes [6-7]. In regards to snoring as a consequence of hypotonia in the muscles of the soft palate, WSM intake should be considered not only for its effective role in increasing muscle mass and strength but also in muscle development and repair.

On the other hand, there is another important key player that should be taken into account regarding sleep: the gut microbiota. Gut microbiota dysbiosis can alter the ventilatory response to hypercapnia, which has been associated with hypercapnic OSAS patients. A study of two rat models with altered gut microbiota showed depressed ventilatory responsiveness to hypercapnia, suggesting that modifications to gut microbiome could induce blunted central chemoreflex control of breathing [8]. This suggests the potential importance of the gut microbiome in ventilatory response, especially in hypercapnic OSAS.

Stress and anxiety are debilitating conditions that can shorten one’s life while reducing performance, well-being, and the overall enjoyment of activities. In addition to the fact that stress can increase inflammation and impair the immune system by lowering resistance to disease and creating the possibility for opportunistic diseases to occur, it has also been demonstrated to be a causative factor in depression. From the whole set of metabolites produced by our gut microbiota, Gamma-aminobutyric acid (GABA) is considered to be the main neurotransmitter with inhibitory effects, and its relation to anxiety and depression disorders is solidly accepted [9].

Interestingly, this consists of a bidirectional relationship between gut microbiota and stress; just as cortisol can reshape and alter the balance of intestinal microbiota, the metabolites and substances produced in our guts have an impact on our brain. This constant dialog between both parts is enclosed within the so-called gut-brain axis [10]. Thus, it is a fact that the adequate manipulation of gut microbiota, via lifestyle interventions such as ingestion of food supplements and appropriate diet, is a potential strategy to face stress disorders and, consequently, improve sleep. Besides, with the growing comprehension of the gut-brain axis, the effects of gut microbiota on sleep are currently of great interest.

A healthy brain and nervous system generate proper brain waves (electrical impulses between neurons) at the adequate time and in the right quantities, while an unbalanced brain, such as the one suffering from stress and anxiety, will be unable to do so. There are five types of waves produced by the brain that are related to stress and sleep, out of which alpha waves, which have a range between 7.5-12 Hz, are the ones to consider [11]. These waves demonstrate slow brain activity in the normal waking state such as relaxation states and light meditation. Since alpha waves are linked with relaxed mental states, many experts believe increasing alpha activity may help reduce stress and anxiety, as well as help people stay relaxed.

The present study aims to elucidate the efficacy of WSM in managing snoring, stress, and improving sleep in healthy adults.

## Materials and methods

Study design

**Figure 1:**
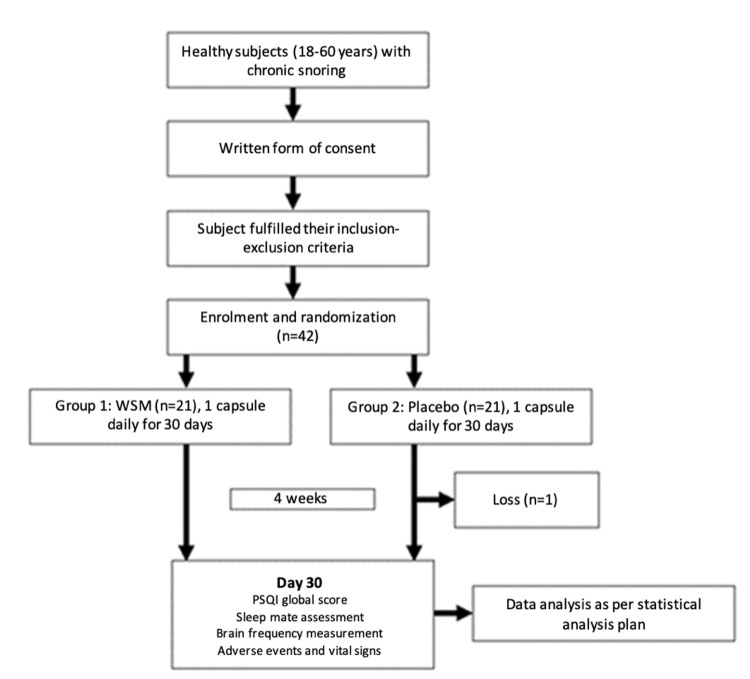
Schematic view of the study design WSM: wild sage metabolites; PSQI: Pittsburgh Sleep Quality Index.

A total of 42 subjects were randomly assigned to one of the two groups, viz. Group 1 (WSM) and Group 2 (Placebo) using a simple randomization process. The primary outcomes measured were changes in Pittsburgh Sleep Quality Index (PSQI) global score and sleep mate’s assessment score, at day 0 and 30. Spontaneously reported and observed adverse events after the first dose until the end of product intake were recorded as secondary outcomes (see Figure 1 for a study flow chart).

Subjects

This study recruited 42 healthy male and female adult subjects who were non-smokers between the ages of 18 to 60 (inclusive), without any known diseases, and with a body mass index (BMI) between 18.0 kg/mg2 and 24.9 kg/mg2. The recruited subjects complied with the following inclusion criteria: age higher than 18 years and presence of chronic snoring. A patient is considered a chronic snorer if his/her bedmate/roommate reported that they snore more than five days per week and this is corroborated by respiratory polygraphy performed in the patient’s own home. The result of the respiratory polygraphy should indicate the presence of snoring for at least 30% of the nocturnal period, with a confirmed diagnosis of mild-to-moderate OSA (5 ≤ apnea-hypopnea index (AHI) < 30) by polysomnography (PSG), and have roommate or bedmate to submit the information. Subjects representing one or more of the following criteria were excluded from participation in the study: high-risk professions and/or controlling dangerous machines; moderate or severe somnolence during the day time; coronary cardiopathy, acute vascular disease (less than three months), chronic and severe obstructive pulmonary disease, and chronic treatment with theophyllines; temporo-mandibular joint problems or periodontitis; mandibular protrusion capacity less than 6 mm and/or less than 10 teeth in each jaw; severe cognitive disorders and/or patients whose answers to the questionnaires are altered by chronic and severe diseases; pregnant women (from the third month of pregnancy to three months after birth delivery).

Test product

WSM is a novel herbal formulation developed by Innovation Labo, Tokyo containing extracts of 100% metabolites from the salvia officinalis leaf. The extract was obtained through two successive steps: (1) Natural fermentation by resting of wild sage with wild sage microbiota for 120 hours at 37°C (2) Extraction to isolate sage metabolites.

Treatment

Test product WSM and Placebo manufactured by Innovation Labo, Tokyo were delivered in pillboxes containing 30 capsules of either WSM or Placebo. The products were labelled with an ID code number. All products were blinded by the sponsor. Each participant was assigned an ID subject number. ID subject numbers were recorded with the ID code numbers of the corresponding two pillboxes for each subject. Supplementation with the assigned study product began after enrolment on day 1 and then continued until day 30. On day 1, the study coordinator gave one pillbox of the product to each subject. On day 30 +/- 1, subjects were asked to come to the study site for clinical examination and brain frequency measurement. The route of administration was oral, once every day before going to sleep for 30 consecutive days with a glass of water (200 mL). The recommended dosage of test product WSM was 450 mg/day. Study subjects were instructed to store the trial products at room temperature in a dry environment, without refrigeration, and away from direct sunlight. Empty pillboxes were recollected on day 30 for monitoring intake compliance and were kept as empty pillboxes for record purposes. During brain frequency measurement, participants were asked to ingest WSM or the Placebo and rest for 30 min before performing brain wave measurement.

Study procedure

This study was conducted in compliance with the Good Clinical Practices Standards, Nuremberg Code, Declaration of Helsinki, Belmont Report. Written informed consent was obtained from each subject. If the subject was unable to read, the subject’s legally acceptable representative was required to be present during the entire informed consent process. The subject’s legally acceptable representative was informed about the study and signed the informed consent form on behalf of the subject once he/she understood and was satisfied with the whole informed consent process. All questions/queries raised about the study were answered to the satisfaction of the subject or the subject’s legally acceptable representative. If a subject or legally acceptable representative was unable to read, an impartial witness was made present during the entire informed consent process. After the written informed consent form and other written information provided to subjects were read and explained to the subject or the subject’s legally acceptable representative, and after the subject or the subject’s legally acceptable representative has orally consented to the subject’s participation in the study, the informed consent form was dated and signed by the subject. 

Demographic details

Demographic details like age, gender, race, height, weight, and BMI were collected at the time of screening (visit 1). 

Measurements

Sleep Quality and Snoring Assessment

Snorers were asked to assess sleep quality by the PSQI [12] and both snorers and sleep mates were asked to grade their condition and their partner’s condition on the following criteria:

• Patient’s snoring reduced

• Patient’s sleep improved

• Patient’s mood improved

• Patient’s fatigue feeling improved

Brain Frequency Measurement

NeuroSky Mindwave mobile device (EEG equipment) (Neurosky, San Jose, CA, USA) was used to measure brain wave frequency.

The brain wave data is collected during the EEG operation and focuses on the alpha frequency band during the resting state, continuously for five minutes. Alpha-rhythm in frequencies between 7.5-12.5 Hz ranges can be detected. Each collected data demonstrates the different levels of alpha waves for the Placebo and treatment group, which were then converted into a voltage. 

Statistical analysis

All categorical data were descriptively represented, while mean ± SD measurements were used for continuous data. For continuous data, distribution was analyzed by normality tests. Parametric or non-parametric tests were applied depending on the type and distribution of data. All statistical procedures used a two-tailed test with a level p < 0.05 considered as statistically significant.

## Results

Basal characteristics of study subjects

Baseline demographics and measurements, viz. age, height, weight, BMI values showed no signiﬁcant (p>0.05) difference between Group 1 (WSM) and Group 2 (Placebo). Demographic characteristics and measurement values at baseline are depicted in Table 1. Of the 42 subjects, 41 were followed until the end of the study. The loss of follow-up occurred for one adult in the Placebo group, in which one person did not return the questionnaire and did not return calls from the investigator.

**Table 1:** Baseline characteristics of study subjects Values are expressed as Mean ± SD.

Parameters	Group 1 – WSM (n=21)	Group 2 – Placebo (n=21)	P value
Age (years)	45.00 ± 2.00	44.00 ± 3.00	>0.05
Height (cm)	165.20 ± 3.90	166.70 ± 4.70	
Weight (kg)	61.30 ± 4.70	59.70 ± 3.50	
BMI (kg/m^2^)	22.50 ± 2.10	21.50 ± 1.20	

Effect of WSM on PSQI score and sleep mate’s assessment score

**Figure 2:**
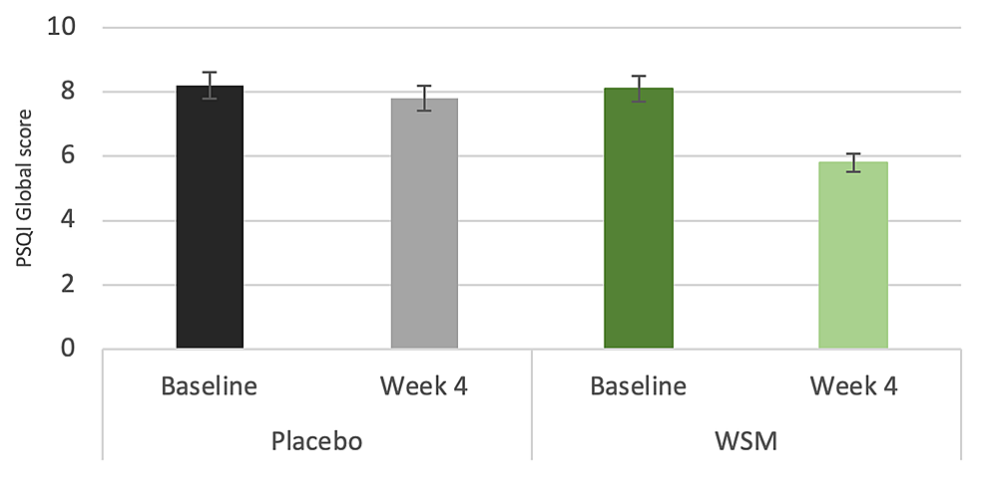
PSQI global score at baseline and at week four of WSM or placebo supplementation PSQI: Pittsburgh Sleep Quality Index; WSM: wild sage metabolites.

After four weeks of WSM supplementation, PSQI global score decreased by 29%. There were no significant changes in the placebo group (Figure 2). Lower values of PSQI global score indicate better sleep. The PSQI global score ranges from 0 to 21.

**Figure 3:**
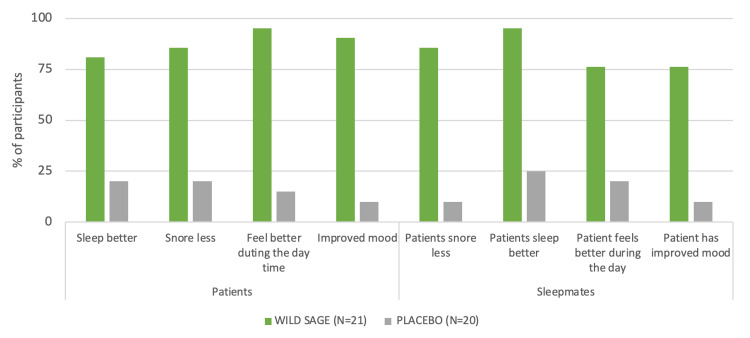
Assessment score at baseline and at week four of wild sage metabolites (WSM) or placebo supplementation

After four weeks of WSM supplementation, assessment scores by patients and sleep mates showed that the majority of participants observed improvement in sleeping, snoring, mood, and the state of health of the patients (Figure 3).

Effect of WSM on brain frequency 

**Figure 4:**
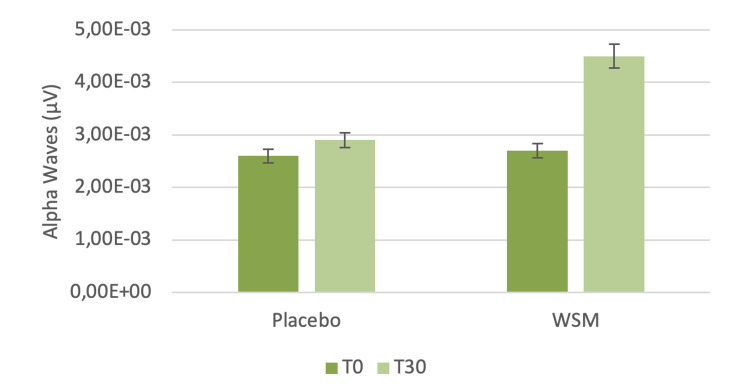
Alpha wave brain frequency at baseline and 30 minutes after wild sage metabolites (WSM) or placebo supplementation

After 30 minutes of WSM supplementation, alpha wave brain frequency increased by 55%. There were no significant changes in the placebo group (Figure 4). 

With regard to safety measures assessment, no cases of expected or unexpected adverse events were reported and no dropouts resulting from adverse events occurred during the study. 

## Discussion

The most important findings of the present study are that the WSM developed by Innovation Labo has been shown to improve sleep scores, sleep quality, snoring, and activation of alpha brain waves. These WSM contain a complex of metabolites from natural fermentation, such as vitamin D, which stimulates muscle cell differentiation. The relevance of such a process regarding sleep quality lies in its role in reducing hypotonia, specifically in the upper airway muscles. As described by Schwartz AR et al. (1998), the structural properties and muscle tone of the region in question are decisive in determining the collapsibility of structures as the soft palate, whose collapsing would mean the obstruction of the airways and thus its consequent reduction of sleep quality [13]. Therefore, having proved that WSM can tighten soft tissue tone, hence preventing soft palate to collapse and obstruct airways, it has been revealed that people suffering from either chronic snoring or, in general, low-quality sleep, might benefit from WSM supplementation.

Apart from the aforementioned, in this study, other mechanisms flattered by WSM supplementation are shown to influence sleep quality. Accordingly, WSM not only improves sleep quality by affecting muscle differentiation and so hypotonia but also acts through modulating gut microbiota. Particularly, WSM potentiates the presence and increases the number of GABA-producing bacteria, which leads to activation of the gut-brain axis. Together with the fact that it is widely accepted that the gut-brain axis is playing a non-insignificant role in wake-sleep regulation, several published studies demonstrated how GABA improves sleep [11,14-15]. Actually, this was not an unexpected fact taking into consideration that GABA is the principal neurotransmitter with inhibitory effects of the central nervous system (CNS), and it is well established that the activation of its receptor favors sleep and reduces stress, reasons why many hypnotics and anti-stress supplements including this molecule in their formulation are available in the market since some years [14-15]. Founded on the same justification, the stress-reduction role of WSM whose outcomes are reflected in the sleep quality as well is, explained. 

Finally, the present study demonstrates that WSM is effective to be used for food applications to manage snoring, improve sleep quality, and reduce stress.

## Conclusions

In conclusion, our study showed that supplementation with WSM at the dose of 450 mg/day resulted in reduced PSQI score and improved both snoring and sleeping assessment by sleep mates after 30 days of treatment, with good tolerance among subjects and without side effects or adverse effects. Accordingly, with the improvement of sleep quality by PSQI score and mate assessment, participants within the WSM group also presented higher levels of alpha waves when compared with placebo. Hence, WSM at 450 mg/day could be recommended as a natural agent to manage snoring, improve sleep quality, and reduce stress by alpha wave activation. Additional studies, particularly mechanistic studies, are warranted to confirm and generalize our results, as well as to acquire a better understanding of how these metabolites may play their role in modulating the gut-brain axis, resulting in the regulation of sleep.
